# Designing eHealth Interventions for Pediatric Emergency Departments: Protocol for a Usability Testing Study With Youth, Parent, and Clinician Participants

**DOI:** 10.2196/64350

**Published:** 2025-04-14

**Authors:** Mari Somerville, Lori Wozney, Allyson Gallant, Janet A Curran

**Affiliations:** 1 IWK Health Halifax, NS Canada; 2 School of Nursing Dalhousie University Halifax, NS Canada

**Keywords:** eHealth intervention, emergency department, usability testing, youth, health services, parents, pediatric, digital health tools, mixed methods, quantitative surveys

## Abstract

**Background:**

Usability tests provide important insight into user preferences, functional issues, and differences between target groups for health interventions and products. However, there is limited guidance on how to adapt the usability testing approach for a youth audience, especially for digital health interventions.

**Objective:**

This protocol paper outlines a novel approach for conducting usability tests with a diverse audience of youth, parents, and clinicians in the development of 2 digital health tools for the pediatric emergency department (ED) setting.

**Methods:**

This paper outlines a protocol for usability testing as part of a broader study aimed at co-designing ED discharge communication tools with youth, parents, and clinicians. The broader study involved co-designing 2 digital tools: one for asthma and one for concussions. A multimethods approach to usability testing was used to assess the functionality of these tools through 2 rounds of testing. A mix of youth, parents, and ED clinicians were invited to participate in each round of usability testing. Participants were asked to provide feedback on the tools through quantitative surveys and open-ended qualitative questions. The usability testing approach was adapted to suit each target group, such as including a youth in the data collection process, to enhance the quality of the data. The severity of usability problems was analyzed following the first round of testing, and each tool was refined based on this feedback. The second round of usability tests involved collecting both qualitative and quantitative feedback on the revised tools.

**Results:**

All usability data have been collected and are being analyzed. Outcomes will be disseminated through a subsequent publication. Results will include demographic characteristics from each user group from both rounds of testing, severity of usability scores, qualitative and quantitative feedback, and differences in test outcomes between each target group.

**Conclusions:**

This paper provides novel guidance for conducting usability tests with youth participants when designing digital health tools. By using a comprehensive co-design and usability testing approach, we anticipate that final tools will be highly relevant to the end users and will lead to better uptake and patient outcomes when pilot-tested in future studies. The outlined approach may be adapted to different health care contexts for other youth participants. Further research should continue to explore ways to design usability tests that are suitable for youth audiences, as there is still a significant gap in the literature around this topic.

**International Registered Report Identifier (IRRID):**

DERR1-10.2196/64350

## Introduction

The International Organization for Standardization describes usability as “the extent to which a system, product or service can be used by specified users to achieve specified goals with effectiveness, efficiency and satisfaction in a specified context of use” [1]. Usability testing is a method in which a product is evaluated by users as they perform tasks, and may include formative or summative testing [2]. Usability testing is considered a cornerstone of user-centered design, valuable for capturing user preferences, to identify any functional issues and determine differences between how certain demographic groups use a product or tool [3]. This information is helpful for both designers and researchers who want to ensure that a product suits the needs of the end user.

In the context of eHealth (ie, digital health tools and health information technologies), applying methods and approaches to usability testing could improve the design and implementation of new interventions. This includes digital tools such as mobile apps, kiosks, virtual care, and electronic health records [4]. The World Health Organization recognizes eHealth interventions as a global priority, as part of the 2020-2025 global strategy on digital health, to create more efficient and effective health care systems [5]. Despite this global health priority setting, it is unclear how often eHealth interventions undergo usability testing prior to implementation. One systematic review reported limited or poor-quality usability testing of electronic health records prior to implementation [6], while another review of 104 eHealth interventions reported that only 38% of them included an aspect of usability testing [4]. Often, eHealth researchers rely on industry-focused protocols, which may lack transferability to complex health care contexts. To evolve the rigor and field of usability testing in the development of eHealth interventions, more diverse testing protocols applied in specific health contexts (eg, emergency departments [EDs]), for specific populations (eg, youth, their caregivers, and clinicians), or for specific types of eHealth interventions (ie, websites versus kiosks) are needed.

Further, eHealth researchers should develop usability protocols that are developmentally appropriate for the target users (ie, age and condition) and relevant to the specific context (ie, hospital or outpatient clinic) where they may be deployed. A scoping review by Maramba et al [7] identified 133 studies where usability testing informed the development of eHealth interventions. However, no studies in the review reported on the usability testing of eHealth interventions in the ED setting, and despite 9 studies being related to child health [7], only 2 studies included youth (aged 14-21 years) as usability testing participants [8,9]. This represents a significant research-to-practice gap as youth and their parents (eg, parents, caregivers, and/or legal guardians) are typically early adopters of eHealth interventions, and their insights could benefit broader adoption. Health services researchers need guidance on how to conduct or adapt usability tests to ensure that end users are appropriately involved in design. The aim of this protocol paper is to describe a youth-, parent-, and clinician-focused approach to usability testing of 2 eHealth interventions for pediatric EDs. This paper will highlight key testing session logistics, considerations for test user eligibility, testing activities and scenarios, adaptations for youth test users, and approaches to synthesizing multimethods usability data.

## Methods

### Study Design

This protocol paper describes one component of the emergency department discharge communication strategies (EDUCATE) study, which aimed to evaluate a co-design method for discharge communication tools for use in the pediatric ED [10]. Based on methodological guidance from Barnum [2] and calls for a more comprehensive approach to usability testing, as reported in previous literature [6], a multimethods approach including quantitative (ie, surveys and severity scoring) and qualitative (ie, open-ended interviews) data was used. Using a multimethods approach allowed us to view usability data from different lenses, with count and frequency data from surveys and experiential data from qualitative sources. The protocol was designed to support remote synchronous usability testing. Remote, synchronous usability testing has previously been shown to be as effective as in-person usability testing for eHealth interventions among both adults and youth [11]. Formative usability testing was used, where tools were evaluated through 2 iterative cycles with a small number of participants [2] to identify any errors prior to implementation. The Template for Intervention Description and Replication (TIDieR) checklist was used to guide the reporting of this protocol paper [12] (Multimedia Appendix 1).

### Ethical Considerations

The study received ethical approval from the institutional review board at IWK Health (#1024004). All participants provided written informed consent prior to each usability test. Additional consent was obtained from a research team member whose image is included in the study materials. Following each usability test, participants received a unique identifier, and their data remained anonymous and confidential. Upon completion of the usability test, all participants received a CAD $30 (US $20.96) gift voucher as a reimbursement for their time.

### Tool Development

During the first phase of the EDUCATE study, 2 electronic discharge communication tools were co-designed by parents, youth, and ED clinicians (ie, nurses and physicians) [13]. A full description of the co-design process will be published in a future publication and is briefly described here. Two co-design teams were established, one for asthma and one for concussion, and met 8 times over a 2-year period between 2020 and 2022. Each co-design team worked together to develop an interactive web-based tool that would address a key discharge communication issue for youth and families visiting the ED. One tool was co-designed to help parents and youth decide whether to visit the ED during an asthma attack, while the second tool was co-designed to help parents and youth navigate the postconcussion recovery journey after leaving the ED. Two user design experts integrated the co-design teams’ ideas into 2 digital tools, which were then assessed for usability.

### Usability Testing Steps

The usability testing process involved four steps, based on usability testing literature [2]: (1) defining the user profiles, (2) the think-aloud process, (3) task-based scenarios, and (4) refining and retesting. Each usability test was facilitated by a researcher trained in mixed and multimethods health services research (MS). Previous literature shows that usability tests often use one approach (ie, quantitative, qualitative, or heuristic methods) to collect usability data, but few use multiple methods [7]. Therefore, a combination of quantitative and qualitative methods was used to gather comprehensive usability data and to identify as many usability issues as possible. The usability tests included a combination of quantitative self-report survey questions, qualitative think-aloud processes, observations, and open-ended interview questions, and were planned to last approximately 60 minutes. This protocol paper focuses on the usability testing process, while more details on recruitment, study setting, and outcome data will be reported in a future publication. Table 1 and the following section describes each of the four usability steps in detail, including how each step was adapted for each target population (ie, youth, parents, and ED clinicians).

**Table 1:** Overview of the usability testing process including each step and corresponding component of the usability test.

Step and item				Goal
**Usability step 1: defining the user profiles**				
	**Presession**			
		Screening questionnaire	Determine eligibility for the usability test	
		Informed consent	Gain written informed consent	
	**Round 1 (total session time: 60 minutes)**			
		Opening script (5 minutes)	Describe the study and provide an overview of tasks Allow participants to ask questions	
		Demographic survey (5 minutes)	Gather demographics, health care usage, and computer skills data	
**Usability step 2: think-aloud process—overview of the “think-aloud” process (5 minutes)**				Describe the think-aloud process Go through 1 live example of the think-aloud process, using a familiar website (all participants) Share a prerecorded video of the think-aloud process with a youth participant (youth participants only)
**Usability step 3: task-based scenarios**				
	Task 1: first impressions (20 minutes)			Gather initial thoughts about the tool Complete a word desirability activity
	Task 2: scenario-based activities (×2; 15 minutes)			Gather qualitative and quantitative metrics related to specific tasks, designed to capture the main functions of the tool
	Gibson survey (5 minutes)			Gather additional quantitative usability testing data
	Thank you and closing remarks (5 minutes)			Thank participants for joining Discuss compensation and next steps
	**Postsession**			
		Quantitative data analysis	Gather time to complete tasks, number and frequency of errors, usability severity scores, and satisfaction data	
		Qualitative data analysis	Gather qualitative feedback about usability issues and satisfaction with the tool	
**Usability step 4: refining and retesting**				
	**Presession**			
		Screening questionnaire	Determine eligibility for the usability test	
		Informed consent	Gain digital e-consent	
	**Round 2**			
		Demographic survey	Gather demographic details about participants prior to testing	
		Posttask questionnaire	Gather quantitative and qualitative usability and satisfaction data on a refined version of the tool through an asynchronous, remote usability test with a new group of end users	

### Step 1: Defining the User Profiles

#### Overview

**Figure 1:**
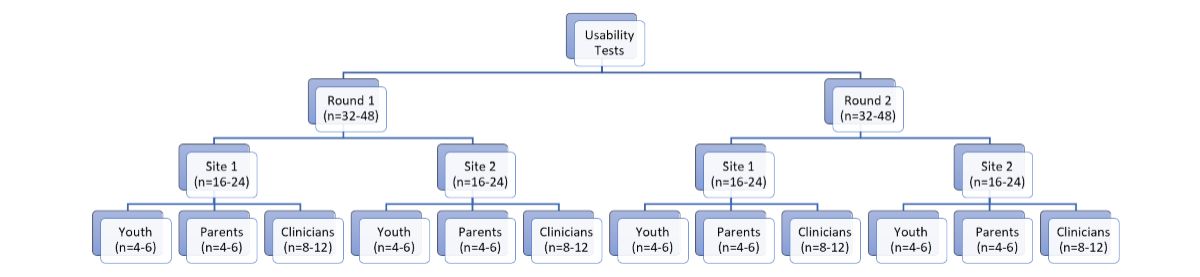
Overview of the expected number of usability testing participants to be recruited from each site for each round of testing for each tool.

Barnum [2] proposes that defining the user profile is an important first step in usability testing. As our tools were codeveloped by and for ED clinicians, parents, and youth, these 3 target groups were chosen as the user profiles. For this study, youth included any individual aged between 12 and 19 years who had visited the ED for either asthma or concussion in the past year. Parent users included any adult (>18 years) who visited the ED with their child for asthma or concussion presentations in the past year, and the clinician profile included any nurse or physician employed in a pediatric ED setting. Nielsen and Landauer [14] argue that 85% of usability issues could be identified with as few as 5 participants, and Barnum [2] proposes that formative usability testing is better suited to a smaller number of participants. Therefore, we aimed to include 2 to 3 participants from each user group (youth, parents, ED nurses, and ED physicians) for each tool across 2 study sites for a proposed sample of 16 to 24 participants from each site in each round of usability testing (Figure 1).

#### Eligibility and Pretest Survey

To determine eligibility, a screening survey was administered to interested participants through the REDCap (Research Electronic Data Capture; Vanderbilt University) platform [15]. Since participants would be testing a digital tool, it was important to screen for health literacy level and access to a computer with audio/video capabilities. To assess health literacy level, the REDCap survey included branching so that parents would be directed to the METER health literacy test [16], which is shown to be a quick and valid measurement of health literacy level among adults. Youth were directed to the Health Literacy Assessment Tool 8 test [17], a quick, feasible and accurate health literacy assessment tool for youth [1]. Clinicians did not have to complete a health literacy test. The screening survey can be found in Multimedia Appendix 2. Participants provided written informed consent and agreed on a day and time to complete a test session with the facilitator (MS).

#### Test Session Setup

Participants joined the session remotely via the Zoom (Zoom Video Communications) using their own computer and webcam. The facilitator provided a brief overview of the study and allowed participants to ask questions to ensure that they understood the expectations of the usability test. The facilitator described that the aim of the usability test was to find problems with the tool and assured participants that their skills and abilities were not being evaluated. This was important to create a safe testing space, particularly for youth participants. Participants were then asked to complete a demographic questionnaire on REDCap prior to the start of the test. The facilitator shared a link to the survey and allowed the participant to complete the test in real time to ensure higher completion rate. Sessions were video recorded.

### Step 2: the Think-Aloud Process

**Figure 2:**
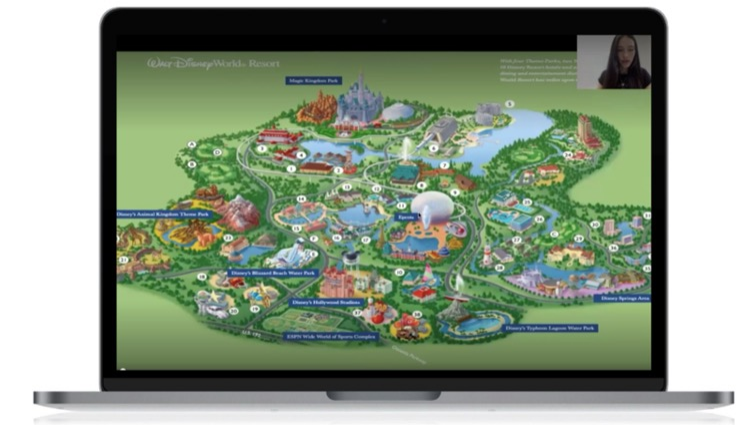
A screen capture of the youth-led think-aloud example.

The think-aloud process involves participants talking through their thought process as they complete a task or solve a problem [18]. This approach is common in usability tests of eHealth interventions [6,7] and is valuable for understanding participants’ decision-making processes rather than strictly observing their behaviors [19,20]. Following the completion of the demographic survey, and immediately prior to the start of the usability test, the facilitator explained to participants how to use a think-aloud approach during the usability test. A mock example was used, which involved navigating a popular Canadian departmental store’s website so that test users could become familiar with the think-aloud process in a web-based environment they recognized. While it was important to demonstrate the think-aloud process with all participants, the co-design team suggested that a second think-aloud example featuring a youth should be modeled for youth participants prior to their usability test. Therefore, a youth member of the co-design team created a 1-minute video of themselves using the think-aloud process to find their way on a popular theme park’s map (Figure 2). This was played for all youth participants prior to the start of the usability test. Examples of the think-aloud process were shared with the co-design teams and refined based on their feedback prior to starting the usability tests with participants.

### Step 3: Task-Based Scenarios

#### Overview

Once participants confirmed that they were comfortable with the think-aloud process, the facilitator started video-recording the usability testing session. The facilitator used a variety of techniques to fully evaluate the usability of the eHealth intervention by adapting traditional usability methods for the end user population (ie, youth, parents, or clinicians). Before sharing a link to the tool, the facilitator shared a link to a web-based Microsoft Word document to guide the user through tasks and included visual prompts to support the scenario-based exercises. This approach was used to eliminate the need for alternating screen sharing by the facilitator and participant and to reduce complexity in the remote testing environment [21]. Six documents were developed, based on the 6 unique user groups (ie, youth with asthma, their parents, and their clinicians; and youth with concussions, their parents, and their clinicians).

#### Task 1: First Impressions

The first task in the usability test was designed to gather participants’ first impressions of the tool. This crucial step in usability testing can quickly determine whether users like or dislike a tool in about 80% of cases [2]. After opening the tool, users were invited to click around, using the think-aloud process to describe their initial reactions to the tool. The facilitator used open-ended prompts to encourage verbalizations of what the participant was thinking; these included the following: “What are your first impressions of the tool?” “What do you think the purpose of the tool is?” Participants were then asked to choose 5-10 words from an adapted list of 118 desirability reaction words of Benedeck and Miner [22]. For usability tests, the Nielsen Norman Group [23] suggests adapting the original list to include approximately 25 words that are appropriate for the user interface being evaluated, with at least 40% of chosen words having a negative connotation. This activity aimed to gather additional user satisfaction data while helping participants, particularly youth, feel more comfortable about sharing their honest thoughts about the tool. This approach has been successfully used in previous usability tests [24]. The list of desirability words can be found in Multimedia Appendix 3.

#### Task 2: Scenario-Based Questions

Next, participants were presented with a scenario relevant to their user identity (ie, youth, parent, or clinician) and medical condition (ie, asthma or concussion). Each scenario was designed by a research team member (MS) based on the user’s persona, as outlined by Quesenbery and Brooks [25]. This involved crafting a situation with the user as the main character where they must achieve a specific goal by using the eHealth intervention being tested. The facilitator used a visual guide and a predetermined script to describe a scenario and then ask participants to complete 2 tasks. The tasks were designed to walk participants through key features of the tool so that additional navigation and usability errors could be easily identified. Participants were asked to use the think-aloud process to describe their thoughts and decisions as they completed each task. Participants were asked open-ended questions about the scenario-based activities, such as the following: “How did you find using the tool to complete that task?” “Is there anything you would change about the tool to make that task easier?” The user-specific tasks are outlined in Multimedia Appendix 4.

#### Global Feedback

Following the scenario-based tasks, participants were asked open-ended questions about the tool, such as the following: “Is there anything else you would change about the tool to make it better?” “On what device/format would you most likely use this tool if you were to use it in the future?” Quantitative data about the functionality and satisfaction of the tool were captured through a REDCap survey, which was administered to participants at the end of the usability test. The facilitator shared a link to the survey using the chat feature of the web-based meeting platform and waited until participants completed the task in real time, to ensure a high completion rate. This posttest was adapted from a survey by Gibson et al [26], which is a validated tool for collecting patient and provider satisfaction data on educational resources. The survey by Gibson et al [26] aims to gather information on visual appeal, functionality, content, and intended use. Branching was used to direct participants to the correct survey in REDCap, as the youth survey also included a question to understand the impact of seeing a youth-led example of the think-aloud process. The posttest surveys adapted from Gibson et al [26] can be found in Multimedia Appendix 5 [26].

### Step 4: Refining and Retesting

Following each usability testing session, the video recording was uploaded to a secure, password-protected internet server. Four coders (MS, JC, AG, LW) watched the videos and independently scored the usability issues using a combination of Nielsen’s [27] scoring system and qualitative analysis. The Nielsen scoring system for severity of usability issues is based on a 5-point scale, ranging from 0 (no usability problem) to 4 (catastrophic usability problem) [27]. Nielsen [27] proposes 3 factors associated with a usability issue: frequency of the problem, impact of the problem, and persistence of the problem. If an eHealth intervention is evaluated to include only minor usability issues (score of 0-2), then the tool may be released without further refinement, while an eHealth intervention with major or catastrophic issues (score of 3-4) should undergo alterations before another round of testing and/or public release [27]. Each coder scored the recorded usability sessions using a deductive approach, following Nielson’s [27] scoring system. A numerical value and descriptive details were entered into an Excel (Microsoft Corp) sheet to explain the reasoning underlying each score. Coders then met to discuss their scores and reach a consensus on final severity scores for each usability test.

In conjunction with Nielson’s [27] severity scoring step, each reviewer made notes about users’ open-ended responses or comments during the usability session. For example, if users described their dislike of a certain feature of the eHealth intervention, the reviewer documented this during the qualitative analysis. Directed content analysis was used to understand the qualitative data and identify the most reported user issues [28]. This type of qualitative analysis allows for a deeper interpretation of qualitative data, often informed by a theory or previous research, and allows for the quantification of the data [28]. In this study, the quantitative findings informed the qualitative data analysis and allowed researchers to calculate the number of user issues with additional context. Following qualitative analysis and severity scoring of each usability test, a description of the most severe usability issues and a list of proposed changes was sent to the design team. The developers then refined the tools by addressing severe usability issues and the most common cosmetic concerns.

**Figure 3:**
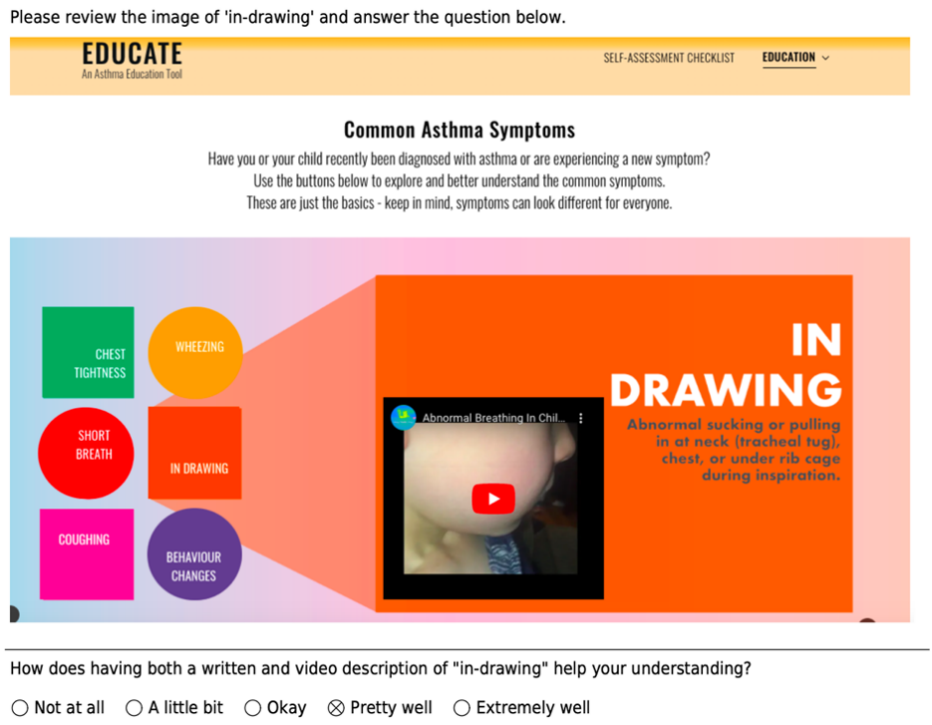
Example of an embedded video clip, along with posttask questions from the asthma tool.

The refined tools were brought to each co-design team for further input before undergoing a second round of usability testing with another sample of target users. As the first round aimed to identify catastrophic usability issues, the second round was intended to identify additional, minor issues. This second round of usability testing involved a modified, remote, asynchronous approach to capture any additional usability issues in the tools. As the first round aimed to identify catastrophic usability issues, the second round was intended to identify additional minor issues. Therefore, a modified approach was used to quickly gather usability information without placing unnecessary burden on participants. To capture remote usability data, images and video clips were embedded in a new REDCap survey to demonstrate the main functions and features of the tools (Figure 3). Participants then completed posttask questionnaires using a Likert scale, informed by Nielson’s [27] methods, with additional free-text boxes to capture qualitative data. Basic demographic questions were also included in the survey.

The final version of each tool was then presented to the co-design team members for their thoughts. Each team made final decisions about what refinements should be incorporated into each tool, signaling the end of the usability testing process.

## Results

The first round of usability testing was conducted between December 2021 and July 2022, while the second round of testing was conducted between November 2022 and March 2023. Data analysis of round 1 took place in July and August 2022 and informed the second round of testing. The final results from both rounds of usability testing will be shared in a future publication. Outcome data will include an overview of severity of usability scores from round 1, qualitative feedback on tool usability and satisfaction from rounds 1 and 2 of testing, and demographic details about study participants. Details about the changes made between rounds 1 and 2 of usability testing will also be presented and may include changes such as button size or location, colors, and new navigation pathways. We will describe any observations related to user characteristics and feedback and identify opportunities for future usability testing and implementation.

## Discussion

### Anticipated Findings

This paper addresses an important gap in the academic usability literature by detailing a co-designed approach to usability testing that was adapted for youth, parents, and clinicians. In particular, this paper describes multiple adaptations that were made to the testing procedures to address developmental stages and comfort levels of youth participants. These adaptations included modeling the think-aloud technique by other youth, allowing for a test-and-try before initiating the recording, screening for the appropriate level of health literacy so participants would be able to complete tasks, using multiple methods for soliciting feedback (self-report survey, observation, and interviews) so participants had varied opportunities to express opinions and suggestions, keeping testing sessions brief and accessible offsite (ie, via Zoom), keeping the sessions short (<60 minutes), using branching logic in data collection methods so participants only accessed information relevant to them, and including less cognitively demanding activities (eg, word desirability activity) to solicit feedback. By applying these approaches to usability testing, it is anticipated that the feedback will be highly relevant, leading to a more user-centered product. We expect that the first round of usability testing will lead to several changes to the tools, while the second round may result in fewer or more minor changes. By using a co-design approach and bringing the usability feedback to each co-design team for further consideration, the next step of piloting the 2 tools in ED settings will lead to positive uptake and outcomes. Previous studies have indicated the benefits of using a co-design approach to engage more end users [29]. However, few usability studies tend to include the youth perspective, even when they are the target audience [7]; hence, we expect this paper to be a significant and beneficial contribution to the usability literature.

### Strengths and Limitations

While this paper provides a comprehensive overview of an approach to usability testing for youth, there are several limitations to consider. Due to the remote nature of the usability tests, participants require internet and computer access. Further, among individuals who do complete a remote usability test, the differences in home environments and technical equipment may affect the quality of the testing process [21]. Future work may focus on offering technical support or request a specific technology setup, as these concerns may have limited participation for some individuals, particularly those from lower socioeconomic backgrounds. Additionally, while it is not a requirement to speak English as a first language to participate in the study, the digital tools were only designed in English, and therefore non–English speakers may be unable to complete the usability tests. Although a small sample of participants is needed to identify most usability issues, a small sample may reduce the generalizability of the findings, which may be seen as a limitation. We have future research planned to mitigate both of these concerns by co-designing multilingual digital tools with broader populations to ensure that the specific needs of clinicians, parents, and youth from varied backgrounds are met. Finally, while the findings of the usability tests may not be generalizable to a non-ED health care context or for individuals presenting with medical conditions apart from concussion and asthma, the techniques used to engage youth may be applied to any usability testing setting.

### Key Recommendations and Conclusion

Youth provide valuable perspectives into eHealth intervention designs and therefore should be included in the usability process; however, there is a significant gap in the literature around usability testing with youth in health services. Therefore, researchers may find the methods used in this paper helpful for guiding usability tests with youth participants in other health care contexts. Further outcome data are needed to determine what works well in youth-based usability studies, some of which will be shared through a future publication presenting the outcomes of the detailed approach.

## Appendix

Multimedia Appendix 1 TIDieR (Template for Intervention Description and Replication) reporting guidelines.

Multimedia Appendix 2 Screening survey for usability participants.

Multimedia Appendix 3 The list of desirability words.

Multimedia Appendix 4 An overview of the user-specific tasks.

Multimedia Appendix 5 Posttest surveys adapted from Gibson et al [27].

## Abbreviations

ED: emergency department
EDUCATE: emergency department discharge communication strategies
REDCap: Research Electronic Data Capture
TIDieR: Template for Intervention Description and Replication

## Data Availability

The datasets generated during this study are not publicly available because the results have not yet been analyzed but are available from the corresponding author on reasonable request.
