# Supplementary material for: Designing eHealth Interventions for Pediatric Emergency Departments: Protocol for a Usability Testing Study With Youth, Parent, and Clinician Participants
Source: JMIR Res Protoc. 2025 Apr 14;14:e64350. doi: 10.2196/64350 (PMC12038285; doi:10.2196/64350)
Supplement: Multimedia Appendix 2 [file resprot_v14i1e64350_app2.docx]

**Multimedia Appendix 2: Screening survey for usability participants**
