# Supplementary material for: Designing eHealth Interventions for Pediatric Emergency Departments: Protocol for a Usability Testing Study With Youth, Parent, and Clinician Participants
Source: JMIR Res Protoc. 2025 Apr 14;14:e64350. doi: 10.2196/64350 (PMC12038285; doi:10.2196/64350)
Supplement: Multimedia Appendix 3 [file resprot_v14i1e64350_app3.docx]

**Multimedia Appendix 3: The list of desirability words**

| **Positive Words** | **Negative Words** |
| --- | --- |
| Clear | Dull |
| Fast | Complex |
| Engaging | Hard to use |
| Useful | Time consuming |
| Helpful | Disruptive |
| Efficient | Annoying |
| Time saving | Slow |
| Effortless | Too technical |
| Valuable | Boring |
| Easy | Stressful |
| Intuitive | Rigid |
| High quality | Unattractive |
| Simplistic | Difficult |
| Effective | Poor Quality |
| Convenient |  |
| Appealing |  |
