# Supplementary material for: Designing eHealth Interventions for Pediatric Emergency Departments: Protocol for a Usability Testing Study With Youth, Parent, and Clinician Participants
Source: JMIR Res Protoc. 2025 Apr 14;14:e64350. doi: 10.2196/64350 (PMC12038285; doi:10.2196/64350)
Supplement: Multimedia Appendix 4 [file resprot_v14i1e64350_app4.docx]

**Multimedia Appendix 4: An overview of the user-specific tasks**

**SCENARIO-BASED QUESTIONS FOR CONCUSSION PARTICIPANTS:**

Concussion Scenario #1: Youth, aged 13 years old, sports player OR non-sports related injury

*Imagine you hit your head during a hockey/rugby/basketball game. You went to the emergency department and the doctor told you that you suffered a concussion. You are given a resource to help you monitor your symptoms over the next few days at home.*

Concussion Scenario #2: Parent of youth, aged 4-5 years old

*Imagine your child fell while at home and hit their head. You took them to the emergency department and were told by a doctor that your child suffered a minor concussion. The doctor or nurse provides you with a tool to monitor your child’s symptoms at home while they recover.*

Concussion Scenario #3: Health care provider (nurse or doctor)

*Imagine you are in the emergency department and have just provided care to a youth with a concussion. In order to support the patient’s post-discharge care, you provide them with a minor head injury management tool. You are extremely busy and only have a short amount of time to provide instructions on how to use the tool. Please complete the following tasks as you would in practice...feel free to pretend that I am the youth or parent of the patient who you are providing instructions to.*

Step 1. Cognitive Analysis Task Approach

Concussion, Parent/Youth

1. Identify where to access the self-assessment score feature. Please enter ‘headache and drowsiness’ as symptoms for the morning of June 22
2. You/your child is vomiting repeatedly and is feeling weak the day after the concussion. Determine your plan of action and whether or not you should return to the emergency dept.

Concussion, Health care provider

1. Instruct the parent/youth how to use the daily tracker component
2. Explain to the parent/youth what symptoms are ‘red flags’ and when to know when to return to the ED, using the tool as a guide

*Please go back to the google document and scroll down to the next page...We created a paper handout to go along with the online tool.*

1. *Can you tell me what you think about this paper version?*
2. *What would make it more useful?*
3. *Can you tell me how you would use this paper handout along with the online version?*

Closing Questions

1. If you were to use this tool in the future, on what device would you most likely use it (phone, computer, ipad)?
2. Can you talk about how you liked or disliked the words used in this tool (eg. Jargon, font size)?

Think Aloud Process – general guiding questions

1. In that scenario what were you thinking/where would you go first/next
2. Please describe what you see in front of you
3. What are you going to do first?
4. What makes you click here/look there?
5. What do you like/dislike about this website/tool?
6. What are you thinking as you are trying to find the answer?
7. Can you describe your thought process for this?

SCENARIO-BASED QUESTIONS FOR ASTHMA PARTICIPANTS:

Asthma Scenario #1: Youth, 16 years old

*Imagine you are at your friend’s house. You start coughing and wheezing and you notice your friend has a dog – you must have been triggered by pet dander. You start to have what feels like an asthma attack. While you are used to experiencing asthma attacks, you are with your friend who doesn’t know how to help you. Your coughing becomes worse and you are having trouble breathing. You feel your chest start to tighten. You wonder if you should go to the emergency department. You remember getting a resource from your GP and consult this resource to decide what to do…*

Asthma Scenario #2: Parent/Coach/Babysitter, caring for young child (e.g., age 5)

*Imagine you are caring for your/a child [aged 5 years], and they start wheezing and have trouble breathing. You are not sure whether you can manage their symptoms with an inhaler or if you should bring them to the emergency department. You consult the resource that you received from a health care provider during a previous visit to help you decide.*

Asthma Scenario #3: Health care provider with low-risk patient

*Imagine you are working in the emergency department and a youth attends with an asthma attack. The attack is minor and only requires inhaler medication, which could have been provided at home. You want to educate the youth/family about asthma symptoms and management so that they can avoid unnecessary trips to the ED in future. You provide them with a resource and walk them through how to use it.*

Step 1. Cognitive Analysis Task Approach

Asthma, Youth/Parent

1. Based on your/your child’s symptoms, determine whether you should head to the emergency department, and what your care plan will be.
2. Overall, how did you find the tool in helping you decide to go to ED?
3. Did you find what you needed to make your decision?
4. Anything we could change/improve to make this better?
5. Using the tool, determine what tracheal tug is, what it looks like and how to identify it (in you and/or your child)
6. How did you find this task of finding tracheal tug?
7. Anything to make this task easier?
8. Did you find everything you needed?

Asthma, Health care provider

1. Provide a five minute education session with the patient showing the different asthma symptoms. Use the education features in the resource to help guide the discussion.
2. Overall how did you find this tool to help with discharge instructions?
3. What would you change about the tool to make this task better/easier?
4. Explain to the patient how they might use this tool in the future to decide whether to go to the ED, based on their symptoms (ie. Use the red, yellow, green light checklist system to explain).
5. How did you find the tool for helping you guide the discussion?
6. Is there anything you would change to make the tool easier/better for showing patients how to decide whether to go to the ED during an asthma attack?

Closing Questions

1. If you were to use this tool in the future, on what device would you most likely use it (phone, computer, ipad)?
2. Can you talk about how you liked or disliked the words used in this tool (e.g., Jargon, font size)

Think Aloud Process – general guiding questions

1. In that scenario what were you thinking/where would you go first/next
2. Please describe what you see in front of you
3. What are you going to do first?
4. What makes you click here/look there?
5. What do you like/dislike about this website/tool?
6. What are you thinking as you are trying to find the answer?
7. Can you describe your thought process for this?
