# Supplementary figures and images for: Designing eHealth Interventions for Pediatric Emergency Departments: Protocol for a Usability Testing Study With Youth, Parent, and Clinician Participants
Source: JMIR Res Protoc. 2025 Apr 14;14:e64350. doi: 10.2196/64350 (PMC12038285; doi:10.2196/64350)

**Multimedia Appendix 5: Adapted Gibson post-test surveys**


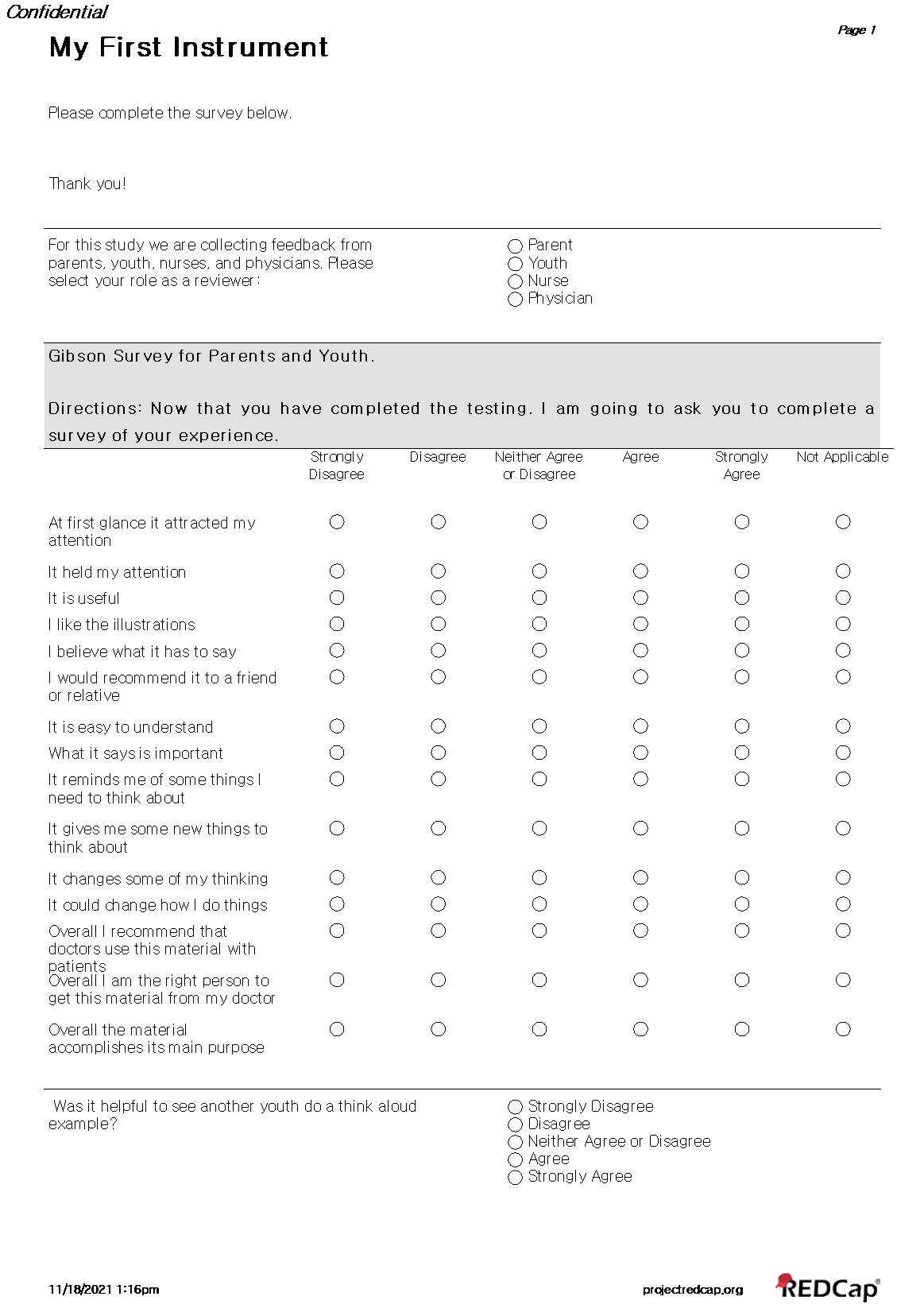

Supplement: Multimedia Appendix 5 [file resprot_v14i1e64350_app5.docx]
